# The role of optic flow pooling in insect flight control in cluttered environments

**DOI:** 10.1038/s41598-019-44187-2

**Published:** 2019-05-22

**Authors:** Julien Lecoeur, Marie Dacke, Dario Floreano, Emily Baird

**Affiliations:** 1 Laboratory of Intelligent Systems, Institute of Microengineering, École Polytechnique Fédérale de Lausanne, Lausanne, CH-1015 Switzerland; 2 Lund Vision Group, Department of Biology, Lund University, Lund, SE-22362 Sweden; 3 Division of Functional Morphology, Department of Zoology, Stockholm University, Stockholm, SE-10691 Sweden

**Keywords:** Computational models, Computer modelling, Animal behaviour, Aerospace engineering

## Abstract

Flight through cluttered environments, such as forests, poses great challenges for animals and machines alike because even small changes in flight path may lead to collisions with nearby obstacles. When flying along narrow corridors, insects use the magnitude of visual motion experienced in each eye to control their position, height, and speed but it is unclear how this strategy would work when the environment contains nearby obstacles against a distant background. To minimise the risk of collisions, we would expect animals to rely on the visual motion generated by only the nearby obstacles but is this the case? To answer this, we combine behavioural experiments with numerical simulations and provide the first evidence that bumblebees extract the maximum rate of image motion in the frontal visual field to steer away from obstacles. Our findings also suggest that bumblebees use different optic flow calculations to control lateral position, speed, and height.

## Introduction

By rapidly processing visual information into motor commands, insects are able to navigate safely in cluttered environments with a level of miniaturisation and refinement that is unmatched by man-made systems. Collision avoidance in clutter is poorly understood in biological systems and is an active field of research for the development of miniature aerial robots^1^ where standard computer vision guidance algorithms require high camera resolution and computing power, which heavily restrict flight time. Understanding and taking inspiration from the simpler–yet effective–techniques employed by insects may help engineers in their quest for miniaturisation and autonomous operation of miniature aerial robots.

Flying insects use the pattern of wide-field image motion on their retina - called optic flow - to control their flight. Optic flow is measured across the panoramic field of view of their compound eyes^2,3^ from the output of arrays of visual motion detection units that extract a motion estimate from neighbouring ommatidia^4^ over a small portion of the visual field. The outputs of these units are then pooled across larger parts of the visual field by integrating neurons^5–8^, which results in a wide-field representation of optic flow that can then be used to guide various aspects of flight behaviour such as lateral position, flight speed, and vertical position. What remains unclear is exactly how the pooling calculation that is being used to control flight is being performed and whether this is done across the entire visual field or in only select parts of it.

When flying through experimental corridors, bees appear to control their lateral position by balancing the magnitude of translational optic flow experienced in the lateral visual field of each eye^9^. Because translational optic flow varies with the inverse of distance^10,11^, this strategy minimises the risk of collision by ensuring that they maintain an equal distance to each wall. Similarly, flying insects also control their flight speed using optic flow–the magnitude of translational optic flow in the lateral, ventral or dorsal visual field^12–14^–which is maintained at a set-point such that, in experimental corridors, flight speed decreases with the distance between the walls^15–18^. It appears that flight speed is regulated by optic flow in the lateral, ventral and dorsal visual fields^12,13,19^. Altitude control in flying insects is much more poorly understood. Bumblebees appear to use optic flow in both the ventral and lateral visual field for vertical position control^20^ but evidence from honeybees suggests that they control altitude by maintaining ventral or dorsal optic flow at a set-point, as for speed control^21,22^. However, without additional input, this control scheme would suffer from a scaling issue – the rate of ventral flow will remain constant if the animal makes equally proportional changes in both speed and altitude. Thus, exactly how insects control their vertical position and where they measure the optic flow used for this remains unclear.

Bees routinely forage in cluttered natural environments, such as around bushes or in forests, where the risk of colliding with obstacles is high. The obstacles that pose the greatest collision risk in clutter are those in front. These would generate higher magnitudes of optic flow than the more distant background, but would subtend only a minor portion of the visual field. If the value for the magnitude of optic flow that is used for flight control is averaged across the output of motion detectors across a broad visual field, then information about the proximity of obstacles in front of the agent will be lost. Thus, how and where in the visual field optic flow is calculated will severely affect the performance of any vision-based flight control and collision avoidance strategies. Previous work provides little insight into how insects measure optic flow for flight control because the pattern of optic flow generated in the experimental corridors that are typically used in these studies is qualitatively independent of viewing angle and the nearest obstacles (the walls) occur only in the lateral visual field.

To investigate how insects control flight in cluttered environments and to identify the optic flow pooling strategies they use to do so, we recorded the trajectories of bumblebees flying through corridors in which the density and placement of obstacles were varied. We then calculated the response of different optic flow pooling methods to our different experimental environments and compared this to the measured data. We find that lateral position and speed control are being regulated by an optic flow pooling strategy that extracts optic flow from nearby obstacles in the frontal visual field but that height control is being regulated by a value of optic flow derived by averaging optic flow across a wide lateral field of view. Overall, our findings suggest that rather than calculating optic flow only across a wide visual field, bees selectively react to nearby obstacles for centring and speed control and that optic flow for different flight control behaviours may be mediated by parallel processing streams in the insect visual system.

## Results and Discussion

**Figure 1:**
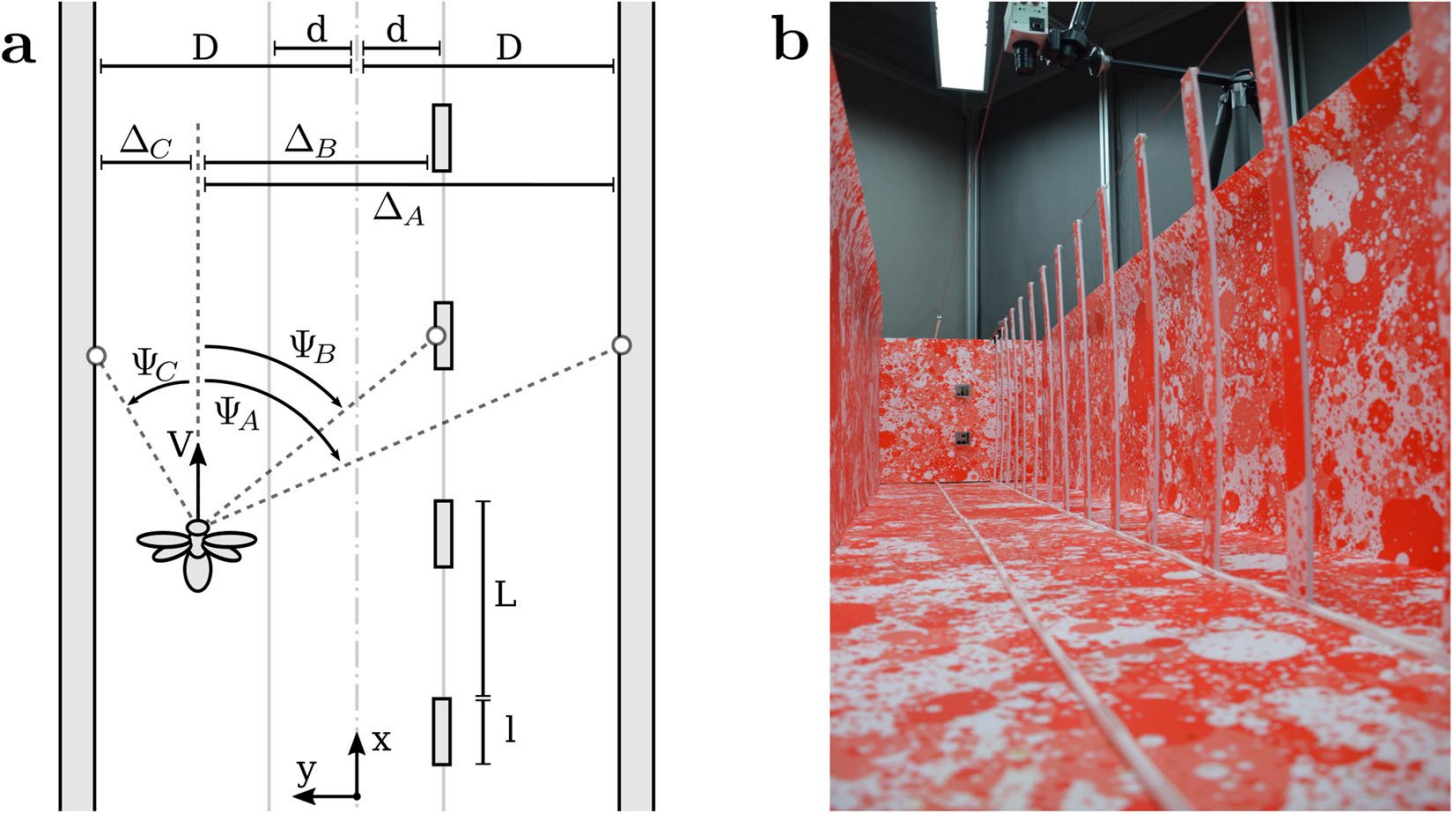
Experimental setting. (**a**) Schematic top view of the experimental corridor. The corridor has the following dimensions: *D* is the half tunnel width, *d* is the lateral position of obstacles, *l* is the obstacle length, *L* is the period at which obstacles are distributed along the corridor. The condition shown here is ${{\mathscr{C}}}_{\mathrm{0|16}}$, with no obstacles on the left (*l*/*L* = 0%), and obstacles occupying 16% on the right side (*l*/*L* = 16%). The state of the agent is modelled by its longitudinal position *x*, its lateral position *y*, its flight speed *V*, and its vertical position *z* (*z* is not shown in this schematic). The viewing angles are defined by the angle Ψ between the *x* axis and a viewing direction. The projection on the *y* axis of the distance between the agent and obstacles is noted Δ and is used for the computation of optic flow in equation [1]. (**b**) View along the experimental corridor with obstacles on the right side.

**Figure 2:**
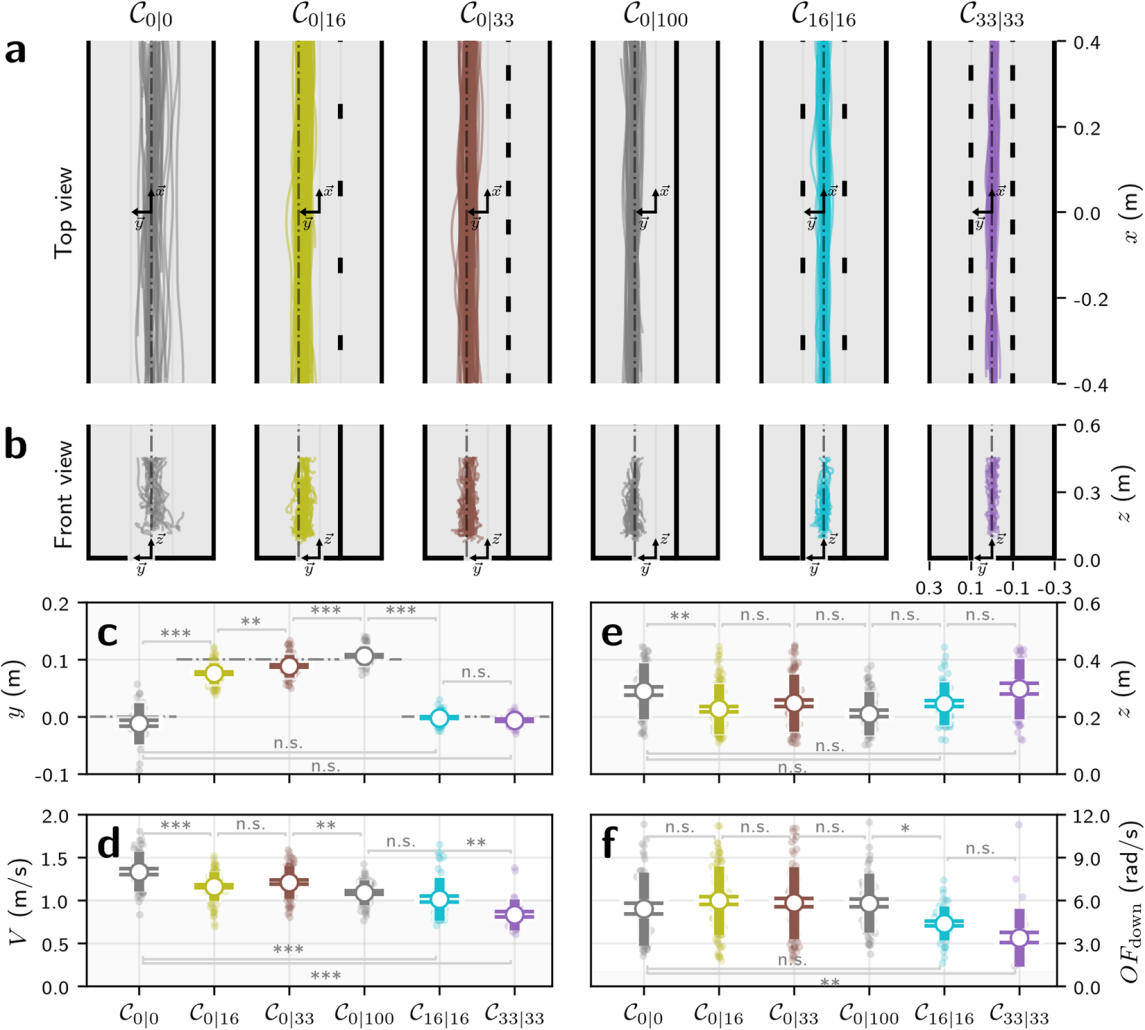
Effect of obstacle density on flight control in bumblebees. In condition ${{\mathscr{C}}}_{\mathrm{0|0}}$, there are no obstacles. In conditions ${{\mathscr{C}}}_{\mathrm{0|16}}$ and ${{\mathscr{C}}}_{\mathrm{0|33}}$, the obstacles are only on one side of the corridor and cover 16% and 33% of its length, respectively. In ${{\mathscr{C}}}_{\mathrm{0|100}}$, the obstacles and the wall on the opposite side form a narrower corridor centred on lateral position *y* = 0.1 m. In conditions ${{\mathscr{C}}}_{\mathrm{16|16}}$ and ${{\mathscr{C}}}_{\mathrm{33|33}}$, the obstacles are on both sides and cover 16% and 33% of the corridor length, respectively. (**a**) Top view of the experimental corridors and recorded trajectories for all conditions. (**b**) Front view of the experimental corridors and recorded trajectories for all conditions. (**c**) Measured lateral positions *y*, expressed in m. (**d**) Measured flight speeds *V*, expressed in m/s. (**e**) Measured vertical positions *z*, expressed in m. (**f**) Ventral flow *OF*_down_, expressed in rad/s, and computed as *OF*_down_ = *V*/*z* from the measured vertical positions and flight speeds. (**c–f**) Measurements are displayed as coloured open circles. The horizontal lines on the error bars denote the standard error of the mean. The uncapped bars denote the standard deviation. The statistical significance of the difference between measured data across two corridor conditions is tested using post-hoc Tukey HSD tests, with the null hypothesis that the mean recorded data is equal for both conditions. The horizontal grey lines indicate the p-value of the post-hoc Tukey HSD tests (*** when *p* ≤ 0.001, ** when 0.001 < *p* ≤ 0.01, * when 0.01 < *p* ≤ 0.05 and *n*.*s*. when 0.05 < *p*).

First, we recorded the three-dimensional position of bumblebees flying along an experimental corridor in which we varied the density of obstacles placed along two lines parallel to the main axis of the corridor at a distance of 0.2 m from each wall. The experimental conditions are named according to the format ${{\mathscr{C}}}_{\alpha |\beta }$ where *α* and *β* represent the percentage of the corridor length occupied by obstacles on the left and right side, respectively, of a bee flying towards a feeder. For example, the corridor ${{\mathscr{C}}}_{\mathrm{0|16}}$ has no obstacles on the left side, and obstacles covering 16% of the tunnel length on the right side (Fig. 1), while in the corridor ${{\mathscr{C}}}_{\mathrm{33|33}}$ obstacles occupy 33% of the corridor length on both sides (Fig. 2a).

### The effect of obstacle density on lateral position

In the conditions without obstacles, ${{\mathscr{C}}}_{\mathrm{0|0}}$ and ${{\mathscr{C}}}_{\mathrm{0|100}}$ (Fig. 2a), bumblebees maintain an equal distance to both walls. With obstacles on one side (conditions ${{\mathscr{C}}}_{\mathrm{0|16}}$ and ${{\mathscr{C}}}_{\mathrm{0|33}}$, Table 1), they instead fly close to the safest position of *y* = 0.1 m (dashed grey line, Fig. 2), but as obstacle density decreases, they fly slightly closer to the obstacles (Fig. 2c).

**Table 1:** Summary of the experimental measurements and statistical significance of the differences measured between pairs of conditions.

		Measurements				Tukey HSD test					
		*μ*	*σ*	*N*	*n*	${{\mathscr{C}}}_{{\bf{0|0}}}$	${{\mathscr{C}}}_{{\bf{0|16}}}$	${{\mathscr{C}}}_{{\bf{0|33}}}$	${{\mathscr{C}}}_{{\bf{0|100}}}$	${{\mathscr{C}}}_{{\bf{16|16}}}$	${{\mathscr{C}}}_{{\bf{33|33}}}$
Lateral Position	${{\mathscr{C}}}_{\mathrm{0|0}}$	−0.012	0.036	46	26		***	***	***	n.s.	n.s.
	${{\mathscr{C}}}_{\mathrm{0|16}}$	0.076	0.018	87	35	0.001		**	***	***	***
	${{\mathscr{C}}}_{\mathrm{0|33}}$	0.088	0.020	73	31	0.001	0.002		***	***	***
	${{\mathscr{C}}}_{\mathrm{0|100}}$	0.107	0.017	50	16	0.001	0.001	0.001		***	***
	${{\mathscr{C}}}_{\mathrm{16|16}}$	−0.002	0.012	49	25	0.163	0.001	0.001	0.001		n.s.
	${{\mathscr{C}}}_{\mathrm{33|33}}$	−0.006	0.011	32	19	0.805	0.001	0.001	0.001	0.900	
Flight Speed	${{\mathscr{C}}}_{\mathrm{0|0}}$	1.337	0.235	46	26		***	**	***	***	***
	${{\mathscr{C}}}_{\mathrm{0|16}}$	1.164	0.171	87	35	0.001		n.s.	n.s.	***	***
	${{\mathscr{C}}}_{\mathrm{0|33}}$	1.212	0.189	73	31	0.010	0.605		**	***	***
	${{\mathscr{C}}}_{\mathrm{0|100}}$	1.091	0.147	50	16	0.001	0.286	0.010		n.s.	***
	${{\mathscr{C}}}_{\mathrm{16|16}}$	1.013	0.249	49	25	0.001	0.001	0.001	0.364		**
	${{\mathscr{C}}}_{\mathrm{33|33}}$	0.836	0.181	32	19	0.001	0.001	0.001	0.001	0.001	
Vertical Position	${{\mathscr{C}}}_{\mathrm{0|0}}$	0.289	0.098	46	26		**	n.s.	***	n.s.	n.s.
	${{\mathscr{C}}}_{\mathrm{0|16}}$	0.226	0.088	87	35	0.002		n.s.	n.s.	n.s.	**
	${{\mathscr{C}}}_{\mathrm{0|33}}$	0.247	0.099	73	31	0.136	0.670		n.s.	n.s.	n.s.
	${{\mathscr{C}}}_{\mathrm{0|100}}$	0.211	0.076	50	16	0.001	0.900	0.251		n.s.	***
	${{\mathscr{C}}}_{\mathrm{16|16}}$	0.245	0.075	49	25	0.177	0.827	0.900	0.412		n.s.
	${{\mathscr{C}}}_{\mathrm{33|33}}$	0.297	0.106	32	19	0.900	0.003	0.097	0.001	0.124	

(Measurements): For each condition (${{\mathscr{C}}}_{\mathrm{0|0}}$, ${{\mathscr{C}}}_{0|16}$, ${{\mathscr{C}}}_{\mathrm{0|33}}$, ${{\mathscr{C}}}_{\mathrm{0|100}}$, ${{\mathscr{C}}}_{\mathrm{16|16}}$ and ${{\mathscr{C}}}_{\mathrm{33|33}}$), *n* bees were recorded while they performed *N* flights through the corridors. For each flight, the lateral position (in m), vertical position (in m), and flight speed (in m/s) were averaged. *μ* and *σ* are the mean and standard deviation across the *N* flights, respectively. (Tukey HSD test): The p-values of post-hoc Tukey HSD tests between values measured in each pair of corridor conditions. The p-values are shown in the entries below the diagonal, and their significance levels are indicated in the corresponding above-diagonal entries. Cases where the null hypothesis that lateral position, vertical position, or flight speed have equal means cannot be rejected are indicated by “n.s.”. The symbols indicate the p-value of the tests: *** when *p* ≤ 0.001, ** when 0.001 < *p* ≤ 0.01, * when 0.01 < *p* ≤ 0.05 and *n:s*: when 0.05 < *p*.

Flights were also centred in corridors with symmetrically distributed obstacles (${{\mathscr{C}}}_{\mathrm{16|16}}$ and ${{\mathscr{C}}}_{\mathrm{33|33}}$), with the lateral position being less variable (lower standard deviations) than in the corridor without obstacles ${{\mathscr{C}}}_{\mathrm{0|0}}$. This is likely due to the factor of 3 reduction in effective corridor width (0.2 m between the obstacles for ${{\mathscr{C}}}_{\mathrm{16|16}}$ and ${{\mathscr{C}}}_{\mathrm{33|33}}$, compared to 0.6 m between the walls in ${{\mathscr{C}}}_{\mathrm{0|0}}$), although the standard deviation decreased by a factor of 3.8 to 4.75 (Table 1). Similarly, even though the maximum width is higher in ${{\mathscr{C}}}_{\mathrm{16|16}}$ and ${{\mathscr{C}}}_{\mathrm{33|33}}$ than in ${{\mathscr{C}}}_{\mathrm{0|100}}$ (0.6 m compared to 0.4 m), the standard deviation of the lateral position is lower in conditions ${{\mathscr{C}}}_{\mathrm{16|16}}$ and ${{\mathscr{C}}}_{\mathrm{33|33}}$ than in conditions ${{\mathscr{C}}}_{\mathrm{0|100}}$. This result suggests that bumblebees respond to the presence of obstacles by increasing the precision with which they control lateral position.

In ${{\mathscr{C}}}_{\mathrm{0|16}}$ and ${{\mathscr{C}}}_{\mathrm{0|33}}$, bumblebees do not fly at an equal distance between the obstacles and the opposite wall, but slightly closer to the obstacles (*p* ≤ 0.001, Table 1). As the density of obstacles increases on one side (0% in ${{\mathscr{C}}}_{\mathrm{0|0}}$, 16% in ${{\mathscr{C}}}_{\mathrm{0|16}}$, and 33% in ${{\mathscr{C}}}_{\mathrm{0|33}}$), bumblebees increase their distance from them (*p* ≤ 0.01, Table 1).

### The effect of obstacle density on flight speed

In the conditions without obstacles (${{\mathscr{C}}}_{\mathrm{0|0}}$ and ${{\mathscr{C}}}_{\mathrm{0|100}}$), bumblebees adjust their flight speed to the corridor width (Fig. 2d). Indeed, speed control in flying insects is often modelled as maintaining the magnitude of optic flow–that is, the ratio between flight speed (*V*) and the distance to nearby surfaces (*D*)–at a predefined value (for a review see^23^), which has been found experimentally to lie between 3.0 rad/s and 6.0 rad/s^17,18,20,24^ for honeybees and bumblebees. In the 0.4 m wide corridor (${{\mathscr{C}}}_{\mathrm{0|100}}$), the average flight speed was 1.1 m/s, which would generate a lateral optic flow of magnitude 5.5 rad/s. In the 0.6 m wide corridor (${{\mathscr{C}}}_{\mathrm{0|0}}$), the average flight speed increased to 1.3 m/s, generating a lateral optic flow of magnitude 4.3 rad/s, representing a speed increase of 20%. This does not match the proportional increase of corridor width, which was 50% and suggests that lateral optic flow is not the only source of information used for flight speed control, a finding that is consistent with the findings of previous studies in both bumblebees^13^ and honeybees^12,17^.

Bumblebees decrease their flight speed from 1.3 m/s in ${{\mathscr{C}}}_{\mathrm{0|0}}$, which is an empty corridor, to 1.0 m/s in ${{\mathscr{C}}}_{\mathrm{16|16}}$, where obstacles cover 16% of the corridor length on each side (*p* ≤ 0.001, Table 1). Flight speed decreases further to 0.84 m/s in ${{\mathscr{C}}}_{\mathrm{33|33}}$ when the density of obstacles increases to 33% on each side (*p* ≤ 0.01, Table 1). With obstacles on only one side of the corridor, speed decreases from 1.3 m/s in ${{\mathscr{C}}}_{\mathrm{0|0}}$ to 1.16 m/s in ${{\mathscr{C}}}_{\mathrm{0|16}}$ (*p* ≤ 0.001, Table 1). Flight speed tends to increase with increasing obstacle density from ${{\mathscr{C}}}_{\mathrm{0|16}}$ to ${{\mathscr{C}}}_{\mathrm{0|33}}$, although not significantly so (*p* = 0.605, Table 1). Finally, speed decreases from 1.21 m/s in ${{\mathscr{C}}}_{\mathrm{0|33}}$, where obstacles cover 33% of the corridor length on one side, to 1.09 m/s in ${{\mathscr{C}}}_{\mathrm{0|100}}$, where the obstacles form a complete wall on one side (*p* ≤ 0.01, Table 1).

Interestingly, bumblebees fly slower when obstacles are present on both sides than when obstacles are present on only one side. They decrease their flight speed from 1.16 m/s in ${{\mathscr{C}}}_{\mathrm{0|16}}$ to 1.01 m/s in ${{\mathscr{C}}}_{\mathrm{16|16}}$ (*p* ≤ 0.001, Table 1) and from 1.21 m/s in ${{\mathscr{C}}}_{\mathrm{0|33}}$ to 0.84 m/s in ${{\mathscr{C}}}_{\mathrm{33|33}}$ (*p* ≤ 0.001, Table 1). This indicates that flight speed is controlled using optic flow on both sides and that it is affected by the presence and density of obstacles.

### The effect of obstacle density on vertical position

In the absence of obstacles, bumblebees maintain the same distance from the floor as they do from the wall: when the half-width of the corridor is 0.3 m (${{\mathscr{C}}}_{\mathrm{0|0}}$) the average vertical position is 0.29 m and, similarly, when the half-width of the corridor is 0.2 m, the average vertical position is 0.21 m. In other words, in corridors that do not contain obstacles, the magnitude of the lateral optic flow is held equal to the magnitude of the ventral optic flow.

In all conditions, the vertical position is quite variable with relatively high standard deviations (Fig. 2e) and there are few cases where the differences between the conditions were significant (Table 1). Interestingly, ventral optic flow (calculated by dividing the measured flight speed by the measured vertical position, Fig. 2f) does not vary between conditions ${{\mathscr{C}}}_{\mathrm{0|0}}$, ${{\mathscr{C}}}_{\mathrm{0|16}}$, ${{\mathscr{C}}}_{\mathrm{0|33}}$, and ${{\mathscr{C}}}_{\mathrm{0|100}}$, but decreases significantly in ${{\mathscr{C}}}_{\mathrm{16|16}}$ and ${{\mathscr{C}}}_{\mathrm{33|33}}$. This is a surprising result because the optic flow profile generated in the ventral visual field is the same for all conditions, so ventral flow should not be affected by the density of obstacles in the lateral visual field. We would expect bumblebees to fly significantly lower in ${{\mathscr{C}}}_{\mathrm{16|16}}$ and ${{\mathscr{C}}}_{\mathrm{33|33}}$ to compensate for the reduced flight speed and to maintain the ventral optic flow at a constant value. However, this is not what we observe (Fig. 2e,f), which suggests that vertical control might not be driven only by ventral optic flow, as previously modelled^21,25–27^, but that it might be mediated by a combination of ventral and lateral optic flow.

### Predicting the effect of obstacle density and optic flow pooling on flight control

The change in lateral position, flight speed, and vertical position in response to the density of nearby obstacles is likely a reflection of the way in which bumblebees pool optic flow from their panoramic field of view to control flight. Are they using a fixed spatial integration of optic flow across their entire visual field or are they selectively reacting to the nearby obstacles? To answer this question, we predict the lateral position, flight speed and vertical position in each of our experimental corridors for different methods of optic flow integration from different parts of the visual field. We then assess how well the results of each method agree with the experimental data from bumblebees to determine which method best explains the observed responses.

### Predicted lateral position

Our predictions of the lateral position are based on the optic flow balancing control strategy in which a sideways force is applied based on the difference between the optic flow on the left and right sides^23^ (for details, see equation [8]). Optic flow on the left and right side are pooled according to four of the most biologically plausible methods: average optic flow across lateral and fronto-lateral visual field (noted respectively avg and avgf), and maximum optic flow in lateral and fronto-lateral visual field (noted respectively max and maxf).

**Figure 3:**
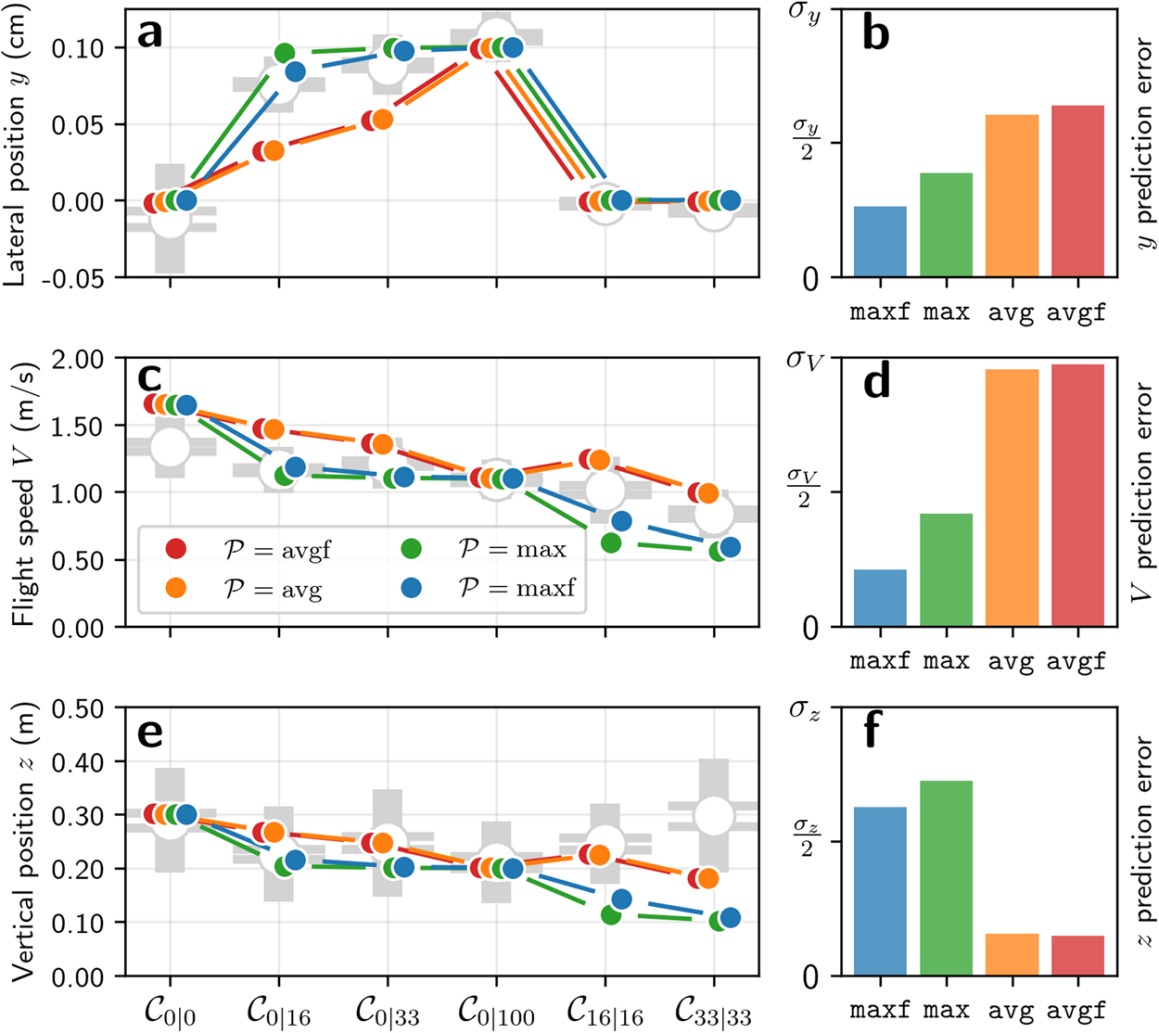
Predicted lateral position, flight speed and vertical position. The experimental measurements of bumblebee lateral position (*y*), flight speed (*V*), and vertical position (*z*) are compared to predicted values. Predictions are made according to the control method described in equation [8], with the same optic flow pooling ${\mathscr{P}}$ on all axes (${\mathscr{P}}={{\mathscr{P}}}_{x}={{\mathscr{P}}}_{y}$). Predictions are made for four optic flow pooling methods avg, avgf, max, and maxf described in equations (2–5). The best prediction for lateral position and flight speed are made with maxf pooling, while the best predictions for vertical position are made with avg and avgf pooling. (**a**) Measured lateral position and predicted lateral position for each experimental condition. (**b**) Average prediction error for lateral position. (**c**) Measured flight speed and predicted flight speed for each experimental condition. (**d**) Average prediction error for flight speed. (**e**) Measured vertical position and predicted vertical position for each experimental condition. (**f**) Average prediction error for vertical position. (**a,c,e**) Measurements are displayed as grey open circles. The horizontal lines on the error bars denote standard error of the mean, the uncapped bars denote the standard deviation. Predictions are displayed as solid coloured circles. Note that predictions are shifted horizontally for clarity. (**b,d,f**) Each prediction is compared with experimental data. The resulting error is scaled according to the standard deviation of the experimental data as shown in equation [14]. This scaling allows comparing prediction errors between conditions where bees controlled their flight with variable precision. The scaled errors are then averaged over conditions ${{\mathscr{C}}}_{\mathrm{0|0}}$, ${{\mathscr{C}}}_{\mathrm{0|16}}$, ${{\mathscr{C}}}_{\mathrm{0|33}}$, ${{\mathscr{C}}}_{\mathrm{0|100}}$, ${{\mathscr{C}}}_{\mathrm{16|16}}$, and ${{\mathscr{C}}}_{\mathrm{33|33}}$.

In the symmetric conditions (${{\mathscr{C}}}_{\mathrm{0|0}}$, ${{\mathscr{C}}}_{\mathrm{0|100}}$, ${{\mathscr{C}}}_{\mathrm{16|16}}$ and ${{\mathscr{C}}}_{\mathrm{33|33}}$), all predictions match the measured lateral positions (Fig. 3a), because the predicted lateral position for these conditions does not depend on the optic flow pooling method and reveals little about the specific optic flow pooling strategy being used. In the asymmetric conditions that have obstacles only on one side (${{\mathscr{C}}}_{\mathrm{0|16}}$ and ${{\mathscr{C}}}_{\mathrm{0|33}}$), the predicted lateral position does vary with the optic flow integration method. It is interesting to note that the predicted lateral positions for average optic flow (avg and avgf) do not vary with changes in the field of view (Fig. 3a). Also, the predictions made using maximum rate of optic flow (max and maxf) have a greater distance to obstacles than those made using averaged optic flow (avg and avgf). This is not surprising because, with the maximum optic flow, the agent selectively reacts to the nearby obstacles, while averaging causes the optic flow from the nearby obstacles to ‘blend’ into the background, reducing their influence on lateral position. The predictions made using the maximum optic flow in the frontal visual field lie the closest to the safest position (at *y* = 0.1 m, i.e. the lateral position at equal distance from the line of obstacles and the opposite wall), and yield lower error than the predictions made using average optic flow when compared to the bumblebee data (Fig. 3b). Optic flow pooling avg and avgf generate lateral positions much closer to the obstacles – and thus much less safe trajectories – than those performed by insects. This suggests that bumblebees use the maximum optic flow in the frontal visual field to control their lateral position, which is the optimal approach because (i) the frontal visual field is where incoming obstacles are the most likely to occur and (ii) selecting the maximum optic flow will ensure that the bees selectively react to the closest obstacles in the visual field.

### Predicted flight speed

Our predictions of flight speed are based on an optic flow regulation strategy in which a longitudinal force derived from the difference between lateral optic flow and a reference optic flow value is applied^20,23^ (for details, see equation [8]). The agent accelerates if the lateral optic flow is lower than the reference optic flow, it decelerates if the lateral optic flow is higher than the reference optic flow, and remains at a constant speed when they are equal. The lateral optic flow is calculated by pooling optic flow on the left and right sides, then taking the mean of the two resulting signals. For the reference optic flow, we use a value of 5.5 rad/s, which is close to the values obtained in previous studies^17,18,20,24^, and is close to the magnitude of lateral optic flow experienced by bumblebees in ${{\mathscr{C}}}_{\mathrm{0|100}}$ (Fig. 2d, Table 1).

In conditions without obstacles (${{\mathscr{C}}}_{\mathrm{0|0}}$ and ${{\mathscr{C}}}_{\mathrm{0|100}}$), the same speed is predicted with all pooling methods (Fig. 3c) and explains why data from simple corridors alone is insufficient for testing hypotheses about how insects pool optic flow for flight control. The predicted speed in ${{\mathscr{C}}}_{\mathrm{0|100}}$ (1.10 m/s) is close to the measured speed (1.09 m/s), which is not surprising given that the reference optic flow value lies close to the optic flow value known to be used by bumblebees. However, the predicted speed for ${{\mathscr{C}}}_{\mathrm{0|0}}$ is 1.65 m/s, which is significantly higher than the measured value of 1.34 m/s (*p* ≤ 0.001). This discrepancy between measured and predicted flight speed in ${{\mathscr{C}}}_{\mathrm{0|0}}$ may be explained by additional sensory feedback – such as airspeed – which is likely combined with optic flow for speed control^28^. However, there is currently no existing model for how airspeed and optic flow are combined to control flight speed in freely-flying insects.

The most interesting conditions for studying the effect of optic flow pooling on flight speed are those in which obstacles are present: ${{\mathscr{C}}}_{\mathrm{0|16}}$, ${{\mathscr{C}}}_{\mathrm{0|33}}$, ${{\mathscr{C}}}_{\mathrm{16|16}}$, and ${{\mathscr{C}}}_{\mathrm{33|33}}$. In these conditions, average pooling avg and avgf generates the highest predicted speeds because maximum pooling max and maxf (Fig. 3c) selects optic flow generated by the closest objects, leading to a reduced flight speed.

The variation of predicted flight speed with varying obstacle density suggests that, as with lateral position control, flight speed is regulated using maximum optic flow pooling. The flight speed predicted using maximum optic flow shows very little variation between ${{\mathscr{C}}}_{\mathrm{0|16}}$ and ${{\mathscr{C}}}_{\mathrm{0|33}}$ (Fig. 3c), which is also the case for the measured flight speeds (Table 1). On the contrary, the flight speed predicted using average optic flow shows a steeper decrease with increasing obstacle density in ${{\mathscr{C}}}_{\mathrm{0|16}}$ and ${{\mathscr{C}}}_{\mathrm{0|33}}$. Furthermore, the predicted flight speed with average pooling is higher in ${{\mathscr{C}}}_{\mathrm{16|16}}$ than in ${{\mathscr{C}}}_{\mathrm{0|100}}$, unlike measured flight speeds which show a non-significant decrease in flight speed (Table 1). The proximity of obstacles in ${{\mathscr{C}}}_{\mathrm{16|16}}$ is higher than in ${{\mathscr{C}}}_{\mathrm{0|100}}$ and it would thus be more sensible to reduce flight speed in this condition, as predicted by maximum pooling.

The predicted speeds lie closer to the measured speeds with maximum pooling than with average pooling (Fig. 3c). The error with maxf pooling is the lowest and is more than 4 times lower than with avgf pooling (Fig. 3d). These results suggest that – similar to lateral control – bees use maximum optic flow pooling in the frontal visual field to control their speed. This makes sense from a biological point of view because it selects the visual motion generated by the closest obstacles, which represent the main collision threats. Also, incoming obstacles are more likely to occur in the frontal visual field, which is coherent with the fact that predictions are more accurate with maxf pooling than with max pooling (Fig. 3d).

### Predicted vertical position

Our predictions of vertical position are based on an optic flow regulation strategy in which a vertical force is applied based on the difference between the ventral optic flow and a reference optic flow value obtained from honeybees^21^ (similar data does not exist for bumblebees; for details, see equation [8]). Note that honeybees use the dorsal flow for controlling vertical position when they are closer to the ceiling than to the ground and ventral flow when flying closer to the ground^22^, we thus consider only the ventral flow in our predictions because the ceiling is located approximately 2 m above the 0.6 m high experimental tunnel. In our test environments, there are no obstacles between the agent and the floor; the ventral optic flow is thus independent of the elevation angle and all optic flow pooling methods provide the same result. We therefore modelled ventral optic flow as the ratio between the flight speed and the vertical position: *OF*_down_ = *V*/*z*, and do not apply pooling.

In the conditions that do not contain obstacles (${{\mathscr{C}}}_{\mathrm{0|0}}$ and ${{\mathscr{C}}}_{\mathrm{0|100}}$), the predicted vertical positions match the measurements (Fig. 3e) and are equal to the corridor half-widths (0.2 m in ${{\mathscr{C}}}_{\mathrm{0|100}}$ and 0.3 m in ${{\mathscr{C}}}_{\mathrm{0|0}}$). This is because flight speed is regulated so that the mean of the pooled optic flow on left and right sides is equal to the reference optic flow value. In obstacle-free corridors, the agent tends to fly at an equal distance from both walls, meaning that pooled optic flow on the left and right sides take the same value, equal to the reference value. Finally, as the vertical position is regulated so that ventral optic flow is equal to the same reference value, the vertical position is equal to the distance between the agent and the walls.

Although no pooling is performed on ventral flow, the predicted vertical position is affected by flight speed, which in turn is affected by the presence of obstacles and thus also the pooling method used. The predicted vertical position is lower when maximum pooling is used on the lateral optic flow than when average pooling is used (Fig. 3e) because the agent flies slower and must reduce its height to maintain ventral optic flow at the reference value.

It is interesting to note that in ${{\mathscr{C}}}_{\mathrm{16|16}}$ and ${{\mathscr{C}}}_{\mathrm{33|33}}$ – i.e. with obstacles on both sides – the predicted vertical positions with max pooling are close to 0.1 m, which is half the distance between the rows of obstacles. Similarly, in ${{\mathscr{C}}}_{\mathrm{0|16}}$ and ${{\mathscr{C}}}_{\mathrm{0|33}}$ – i.e. with obstacles on one side only – the predicted vertical positions with max pooling are close to 0.2 m. Thus, when it comes to vertical control with maximum pooling, the agent acts as if the row of obstacles were a wall, which is not consistent with the behaviour of bumblebees (Fig. 2d,e).

The predictions that best match the experimental data are with average pooling, which yield prediction errors (Fig. 3f) that are approximately 5 times lower than with maximum pooling, suggesting that bees use average pooling of lateral optic flow to control their vertical position.

### Combining multiple optic flow pooling methods for different aspects of flight control

Our model predictions suggest that bumblebees use maximum optic flow pooling in the frontal visual field to control both their lateral position and flight speed but that vertical position is controlled by average optic flow pooling in the lateral visual field. An agent using only maxf would correctly replicate bumblebee lateral position and flight speed, but would not fly at the same vertical position and an agent using only average pooling would correctly replicate bumblebee vertical position, but would not fly at the same lateral position and flight speed. How can we reconcile this contradiction?

Given the parallel nature of neural systems, the same piece of information can be processed by several circuits in the brain, each circuit implementing a different function. For example, the optomotor response and centring behaviour are known to be mediated by two distinct movement detecting pathways in the honeybee visual system^29^. Thus, optic flow from a wide field of view may be processed (or pooled) several times in parallel according to different functions – like average and maximum pooling – and across different visual fields before it is used to control different aspects of flight.

To test this hypothesis, we generated predictions using different optic flow pooling methods in parallel. Namely, in the formulation of the three forces driving the control of lateral position, flight speed and vertical position (equation [8]), we allowed different pooling methods to be used for each axis. While we applied the same control strategies for lateral position and flight speed as before, we modified the control strategy for vertical position. Instead of regulating the ventral optic flow so that it is equal to a fixed reference value, we regulate it according to a value generated from the lateral optic flow (and therefore coupled to flight speed, see equation [9]).

**Figure 4:**
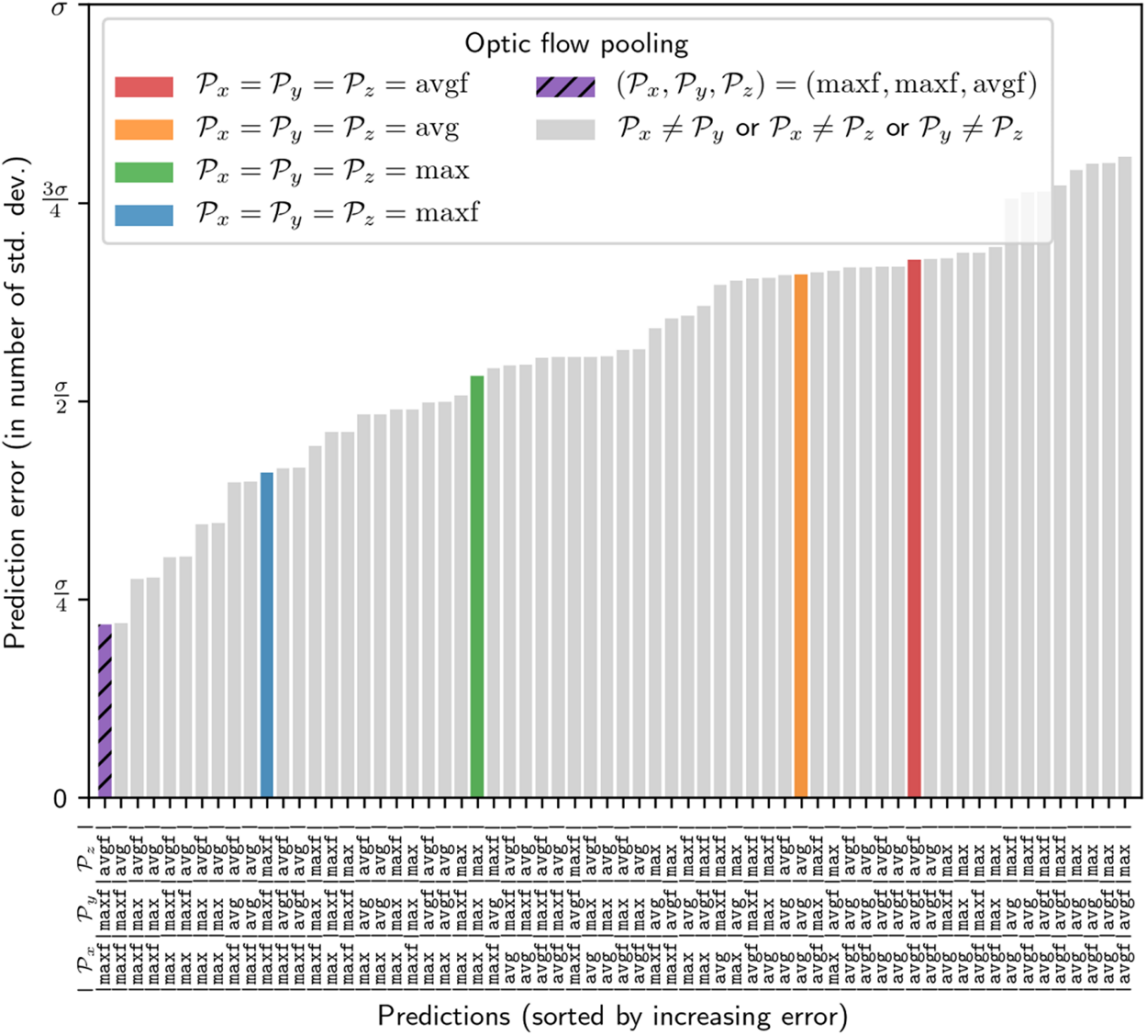
Prediction error when combining multiple optic flow pooling methods, with coupled forward and vertical control. Average prediction errors when different optic flow pooling methods are used to control different aspects of flight. All combinations of optic flow pooling avg, avgf, max, and maxf are tested for the control of lateral position, flight speed and vertical position. For each combination, the prediction error on each axis is computed according to equation [14], then combined into a single error according to equation [13]. The predictions are made according to the control method shown in equation [9], where the reference optic flow used for vertical control is not constant but is computed from the lateral optic flow. The predictions are made using different pooling ${{\mathscr{P}}}^{x}$, ${{\mathscr{P}}}^{y}$, and ${{\mathscr{P}}}^{z}$ for the control of flight speed, lateral position, and vertical position, respectively. Contrary to the uncoupled case (see Supplementary Fig. S1), the best prediction is achieved when combining different optic flow pooling methods. With maximum optic flow pooling in the frontal visual field for forward and lateral control (${{\mathscr{P}}}_{x}={{\mathscr{P}}}_{y}$ = maxf) and average optic flow pooling in the frontal visual field for vertical control (${{\mathscr{P}}}_{z}$ = avgf), the prediction error is the lowest, and is 50% smaller than with ${{\mathscr{P}}}_{x}={{\mathscr{P}}}_{y}={{\mathscr{P}}}_{y}$ = maxf.

**Figure 5:**
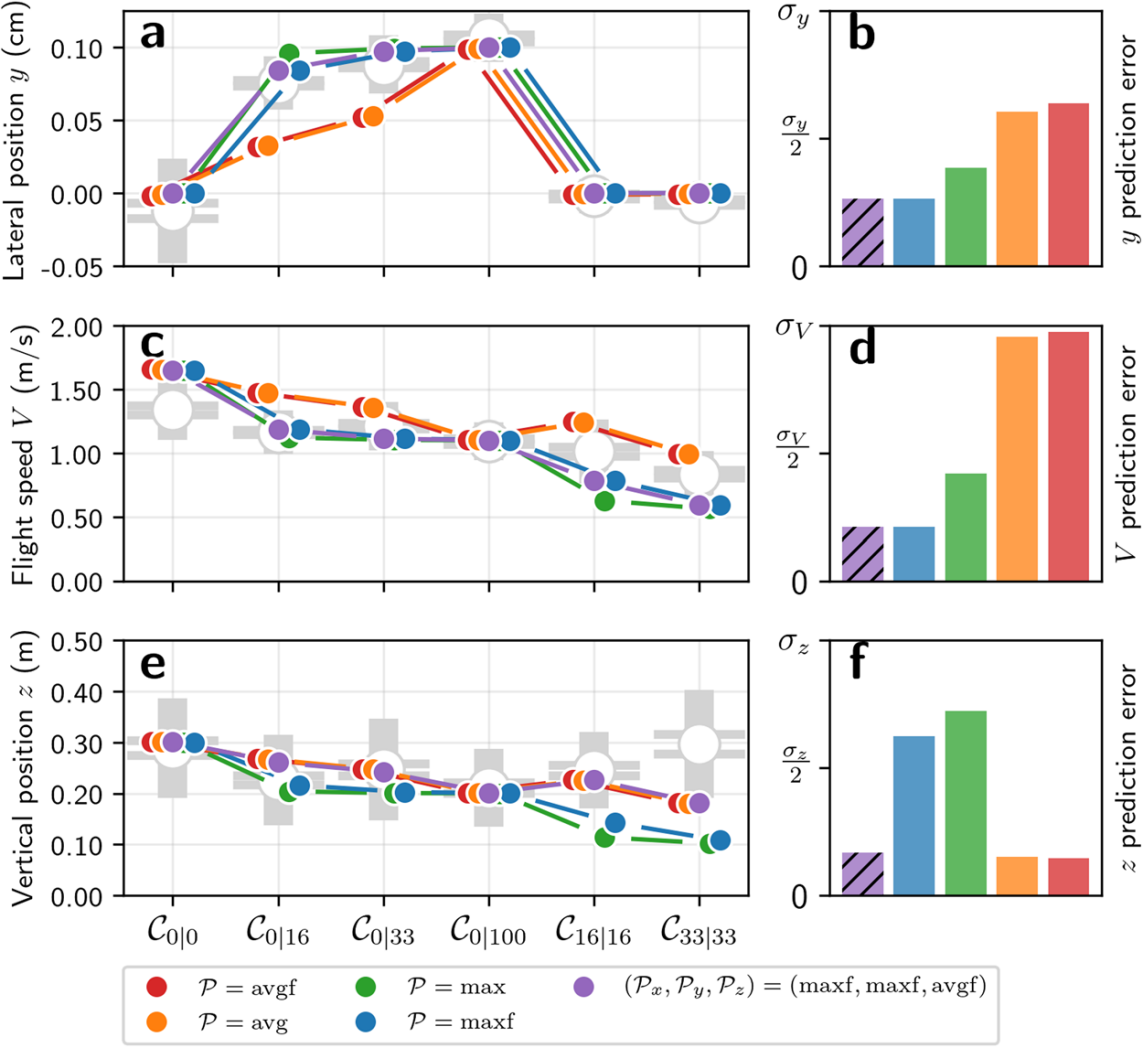
Predicted lateral position, flight speed and vertical position with coupled forward and vertical control. The experimental measurements of bumblebee lateral position (*y*), flight speed (*V*), and vertical position (*z*) are compared to predicted values. Predictions are made according to the control method described in equation [9], where the control of flight speed and vertical position are coupled, and different pooling methods can be combined. For more clarity, all the combinations of optic flow pooling are not presented here. The same pooling is used on all control axes for all presented predictions except one (shown in dashed purple), which yields the lowest prediction error. The lowest prediction error is achieved with maximum optic flow in frontal visual field for lateral and forward control (${{\mathscr{P}}}_{x}={{\mathscr{P}}}_{y}$ = maxf), and average pooling in frontal visual field for vertical control (${{\mathscr{P}}}_{z}$ = avgf). (**a**) Measured lateral position and predicted lateral position for each tested conditions. (**b**) Average prediction error for lateral position. (**c**) Measured flight speed and predicted flight speed for each tested conditions. (**d**) Average prediction error for flight speed. (**e**) Measured vertical position and predicted vertical position for each tested condition. (**f**) Average prediction error for vertical position. (**a,c,e**) Measurements are displayed as grey open circles. As in Fig. 2, the horizontal lines on the error bars denote standard error of the mean. The uncapped bars denote the standard deviation. Predictions are displayed as solid coloured circles. Note that predictions are shifted horizontally for better presentation. (**b,d,f**) Each prediction is compared with experimental data. The resulting error is scaled according to the standard deviation of the experimental data as shown in equation [14]. This scaling allows comparing prediction errors between conditions where bees controlled their flight with variable precision. The scaled errors are then averaged over conditions ${{\mathscr{C}}}_{\mathrm{0|0}}$, ${{\mathscr{C}}}_{\mathrm{0|16}}$, ${{\mathscr{C}}}_{\mathrm{0|33}}$, ${{\mathscr{C}}}_{\mathrm{0|100}}$, ${{\mathscr{C}}}_{\mathrm{16|16}}$, and ${{\mathscr{C}}}_{\mathrm{33|33}}$.

We tested all combinations of optic flow pooling (${{\mathscr{P}}}_{x}$, ${{\mathscr{P}}}_{y}$, ${{\mathscr{P}}}_{z}$) and compared the prediction accuracy. While using multiple optic flow pooling methods did not improve the accuracy of our predictions when the control of speed and vertical position were uncoupled (equation [8] and Supplementary Fig. S1), the predictions were more accurate when the control of flight speed and vertical position were coupled (equation [9] and Fig. 4). Interestingly, the best prediction is achieved with maximum optic flow pooling in the frontal visual field for speed and lateral control (${{\mathscr{P}}}_{x}={{\mathscr{P}}}_{y}$ = maxf) and average optic flow pooling in the frontal visual field for vertical control (${{\mathscr{P}}}_{z}$ = avgf), see the dashed purple bars on Figs 4 and 5.

With ${{\mathscr{P}}}_{x}={{\mathscr{P}}}_{y}$ = maxf and ${{\mathscr{P}}}_{z}$ = avgf, the predicted lateral position is the same as when maxf pooling is used on all axes (purple and blue dots on Fig. 5a). The predicted flight speed is the same as when maxf pooling is used on all axes (purple and blue dots on Fig. 5c). The predicted vertical position is the same as when avgf pooling is used on all axes (purple and red dots on Fig. 5e). Furthermore, the contradiction described earlier is solved because the prediction made with ${{\mathscr{P}}}_{x}={{\mathscr{P}}}_{y}$ = maxf and ${{\mathscr{P}}}_{z}$ = avgf achieves the lowest prediction error on all axes.

## Conclusion

In this study, we investigate the effect of clutter on flight control in bumblebees and use mathematical models to understand how and where optic flow is being measured for this. We find that lateral position is controlled by balancing the maximum optic flow in the frontal visual field. This would ensure that even small obstacles in the flight path will be detected and used to control position, enabling rapid and effective collision avoidance responses. For speed control, the same pooling method is used to calculate the optic flow value that is then compared to a reference value, presumably set by the optimum sensitivity of specific speed-regulating neurons. Detecting the maximum optic flow output across an array of motion detectors is a biologically plausible operation which could easily be implemented in neural systems using a Winner-Take-All network^30^ or using differentiation and zero-crossing^31^, and is supported by previous behavioural^32^ and analytical^33^ studies. Interestingly, we find that bees are most likely using a different pooling method for controlling vertical position. Instead of adjusting their height to maintain the ventral optic flow at a fixed reference value, as suggested for honeybees^21^, we find that bumblebees regulate the ventral flow to a non-constant reference value equal to the average optic flow in the fronto-lateral visual field. It is possible that this finding is specifically related to our experimental situation, which contained vertical obstacles that could not be avoided by changing vertical position. It would be interesting for future investigations to examine the responses to horizontally-oriented obstacles and to model the pooling of lateral and ventral optic flow across varying azimuth and elevation angles. Taken together, our results suggest that bumblebees pool optic flow from the frontal visual field using two methods in parallel - averaging and maximum pooling - to control different aspects of flight. This has important implications for the design of flying robots because our findings suggest that a single forward pointed camera covering the frontal visual field is sufficient to replicate bumblebee behaviour.

## Materials and Methods

### Experimental setting

Bumblebees (*Bombus terrestris*) were trained to fly along an experimental corridor between their nest and a food source. Bumblebees accessed the corridor through small holes and neither the nest nor the food source were visible from the inside. The experimental corridor was 3 m long, 0.6 m wide with 0.6 m high walls (Fig. 1). Each row of obstacles was located *d* = 0.1 m off the center-line of the experimental tunnel. The obstacles consisted of 0.03 m × 0.004 m × 0.6 m vertical plates and were equally distributed along the tunnel. The spacing between the obstacles was varied between infinity (no obstacles), 0.15 m, 0.06 m, and 0 m (a wall without gap).

The walls, floor, and obstacles were covered with a red and white pattern (to improve detection of the bee in the camera images) providing visual contrast in all orientations and thus enabling the insects to measure image motion around them^13,33–35^. This pattern – called dead leaves – has the same frequency content as natural images and is rotation and scale invariant. The scale invariance of the dead leaves pattern implies that nearby obstacles appear with the same frequency content as the more distant background. This ensures that the only way to distinguish the obstacles from the background is the difference of their relative motion. Obstacles can thus be distinguished from the background based on dynamic cues but not based on static cues.

Flights to the feeder were filmed with two high-speed cameras at 200 fps and the 3D trajectories reconstructed. The flight trajectories were mostly rectilinear along the *x* axis, with a constant speed *V* and altitude *z*, and minor oscillations along the lateral *y* axis. We therefore model bee trajectories as straight trajectories at a constant speed, parallel to the *x* axis.

### Geometrical expression of translational optic flow

The magnitude of optic flow perceived by an agent when moving along an experimental corridor can be predicted geometrically^10,11^ according to equation [1].

1 $$\Vert OF(x,y,z,V,{\rm{\Psi }})\Vert =\frac{V\,{\sin }^{2}({\rm{\Psi }})}{{\rm{\Delta }}}$$

where *x*, *y* and *z* are the longitudinal, lateral, and vertical positions of the agent in the corridor, respectively. The lateral position is equal to 0 when the agent is in the center of the corridor and *y* > 0 when the agent is offset towards the left side of the corridor. The vertical position *z* is equal to zero when the agent touches the ground and *z* > 0 when the agent is above the ground. *V* is the forward velocity. Ψ is the viewing angle at which optic flow is measured. The angle Ψ is equal to zero for the viewing direction pointed straight ahead, and Ψ > 0 when looking on the left side of the agent. Δ is the projection onto the *y* axis of the distance between the agent and the closest object (see Fig. 1). In our experimental corridors (conditions ${{\mathscr{C}}}_{\mathrm{0|0}}$ to ${{\mathscr{C}}}_{\mathrm{33|33}}$), the distance Δ can be the distance from the agent to the left wall, right wall, left line of obstacles, or right line of obstacles (Fig. 1).

### Optic flow pooling

We define optic flow pooling as the operation ${\mathscr{P}}$ that consists in computing an optic flow value $O{F}^{{\mathscr{P}}}(x,y,z,V)$ that does not depend on the viewing direction Ψ from a panoramic optic flow field *OF*(*x*, *y*, *z*, *V*, Ψ). We consider four pooling methods, noted ${{\mathscr{P}}}_{{\rm{avg}}}$, ${{\mathscr{P}}}_{{\rm{avgf}}}$, ${{\mathscr{P}}}_{{\rm{\max }}}$ and ${{\mathscr{P}}}_{{\rm{maxf}}}$, that differ in the extent of the field of view (either complete or frontal field of view) and in their pooling function (either average or maximum pooling) as described below.

The average optic flow pooling used in this paper is noted ${{\mathscr{P}}}_{{\rm{avg}}}$ and computed according to equation [2] for viewing angles Ψ ∈ [0, *π*] and Ψ ∈ [−*π*, 0].

2 $$\begin{array}{rcl}O{F}_{{\rm{left}}}^{{{\mathscr{P}}}_{{\rm{avg}}}}(x,y,z,V) & = & \frac{2}{\pi }{\int }_{0}^{\pi }\Vert OF(x,y,z,V,{\rm{\Psi }})\Vert .\,d{\rm{\Psi }}\\ O{F}_{{\rm{right}}}^{{{\mathscr{P}}}_{{\rm{avg}}}}(x,y,z,V) & = & \frac{2}{\pi }{\int }_{-\pi }^{0}\Vert OF(x,y,z,V,{\rm{\Psi }})\Vert \mathrm{.}d{\rm{\Psi }}\end{array}$$

The average optic flow pooling in the frontal visual field is noted ${{\mathscr{P}}}_{{\rm{avgf}}}$ and computed according to equation (3) for viewing angles Ψ ∈ [0, *π*/2] and Ψ ∈ [−*π*/2, 0].

3 $$\begin{array}{rcl}O{F}_{{\rm{left}}}^{{{\mathscr{P}}}_{{\rm{avgf}}}}(x,y,z,V) & = & \frac{4}{\pi }{\int }_{0}^{\pi \mathrm{/2}}\Vert OF(x,y,z,V,{\rm{\Psi }})\Vert \mathrm{.}d{\rm{\Psi }}\\ O{F}_{{\rm{right}}}^{{{\mathscr{P}}}_{{\rm{avgf}}}}(x,y,z,V) & = & \frac{4}{\pi }{\int }_{-\pi \mathrm{/2}}^{0}\Vert OF(x,y,z,V,{\rm{\Psi }})\Vert \mathrm{.}d{\rm{\Psi }}\end{array}$$

The maximum optic flow pooling is noted ${{\mathscr{P}}}_{{\rm{\max }}}$ and computed according to equation (4) for viewing angles Ψ ∈ [0, *π*] and Ψ ∈ [−*π*, 0].

4 $$\begin{array}{rcl}O{F}_{{\rm{left}}}^{{{\mathscr{P}}}_{{\rm{\max }}}}(x,y,z,V) & = & \mathop{{\rm{\max }}}\limits_{{\rm{\Psi }}\in [0,\pi ]}\Vert OF(x,y,z,V,{\rm{\Psi }})\Vert \\ O{F}_{{\rm{right}}}^{{{\mathscr{P}}}_{{\rm{\max }}}}(x,y,z,V) & = & \mathop{{\rm{\max }}}\limits_{{\rm{\Psi }}\in [-\pi \mathrm{,0]}}\Vert OF(x,y,z,V,{\rm{\Psi }})\Vert \end{array}$$

The maximum optic flow pooling in the frontal field of view is noted ${{\mathscr{P}}}_{{\rm{maxf}}}$ and computed according to equation (5) for viewing angles Ψ ∈ [0, *π*/2] and Ψ ∈ [−*π*/2, 0].

5 $$\begin{array}{rcl}O{F}_{{\rm{left}}}^{{{\mathscr{P}}}_{{\rm{maxf}}}}(x,y,z,V) & = & \mathop{{\rm{\max }}}\limits_{{\rm{\Psi }}\in [0,\pi /2]}\Vert OF(x,y,z,V,{\rm{\Psi }})\Vert \\ O{F}_{{\rm{right}}}^{{{\mathscr{P}}}_{{\rm{maxf}}}}(x,y,z,V) & = & \mathop{{\rm{\max }}}\limits_{{\rm{\Psi }}\in [-\pi /2,\mathrm{0]}}\Vert OF(x,y,z,V,{\rm{\Psi }})\Vert \end{array}$$

Ventral optic flow is modelled as the ratio between flight speed and vertical position:

6 $$O{F}_{{\rm{down}}}(x,y,z,V)=\frac{V}{z}$$

### Force field for flight control

The optic flow balancing behaviour of bees is here modelled as a force field. The force field $f\,:{ {\mathcal R} }^{4}\to { {\mathcal R} }^{3}$ defines three forces *f*_*x*_, *f*_*y*_ and *f*_*z*_ for every agent state (*x*, *y*, *z*, *V*).

7 $$\overrightarrow{f}(x,y,z,V)=[\begin{array}{c}{f}_{x}(x,y,z,V)\\ {f}_{y}(x,y,z,V)\\ {f}_{z}(x,y,z,V)\end{array}]$$

The forces *f*_*x*_, *f*_*y*_ and *f*_*z*_ respectively drive the agent along the *x* axis (i.e. controls flight speed), the *y* axis (i.e. controls the lateral position), and the *z* axis (i.e. controls the vertical position).

#### Force field for predictions of lateral position, vertical position, and flight speed

The predictions with uncoupled forward and vertical control (Fig. 3 and Supplementary Fig. S1) are made using the force field presented in equation [8].

8 $$\begin{array}{rcl}{f}_{x} & = & O{F}_{{\rm{ref}}}-\frac{O{F}_{{\rm{left}}}^{{{\mathscr{P}}}_{x}}+O{F}_{{\rm{right}}}^{{{\mathscr{P}}}_{x}}}{2}\\ {f}_{y} & = & O{F}_{{\rm{right}}}^{{{\mathscr{P}}}_{y}}-O{F}_{{\rm{left}}}^{{{\mathscr{P}}}_{y}}\\ {f}_{z} & = & O{F}_{{\rm{down}}}-O{F}_{{\rm{ref}}}\end{array}$$

where ${{\mathscr{P}}}_{x}$ and ${{\mathscr{P}}}_{y}$ are the optic flow pooling methods used for lateral control and speed control, $O{F}_{{\rm{left}}}^{{\mathscr{P}}}$ and $O{F}_{{\rm{right}}}^{{\mathscr{P}}}$ are the optic flow pooled using the pooling method ${\mathscr{P}}$ on the left and right side, according to equations (2–5), *OF*_down_ is the ventral optic flow computed according to equation [6], and *OF*_ref_ is a constant reference value. Note that this commonly accepted control scheme – although valid in a constrained environment like the one used in this study – would suffer from a scaling issue in an open, flat environment. Indeed, without objects on the sides, the lateral optic flow would be null and the agent would accelerate forward and upward infinitely, trying to match the reference *OF*_ref_. In order to solve this inconsistency, our study may be extended to include viewing directions pointed slightly above and below the horizontal plane into the calculations of the lateral optic flow, in which case the ground would remain visible in a portion of the lateral visual field when flying in an open environment. Furthermore, bumblebees are likely to rely on a additional control mechanism involving the measurement of airspeed; this is, however, outside of the scope of our study.

#### Force field for predictions with coupled vertical and forward control

The predictions with coupled forward and vertical control (Figs 5 and 4) are made using the force field presented in equation [9].

9 $$\begin{array}{rcl}{f}_{x} & = & O{F}_{{\rm{ref}}}-\frac{O{F}_{{\rm{left}}}^{{{\mathscr{P}}}_{x}}+O{F}_{{\rm{right}}}^{{{\mathscr{P}}}_{x}}}{2}\\ {f}_{y} & = & O{F}_{{\rm{right}}}^{{{\mathscr{P}}}_{y}}-O{F}_{{\rm{left}}}^{{{\mathscr{P}}}_{y}}\\ {f}_{z}^{\ast } & = & O{F}_{{\rm{down}}}-\frac{O{F}_{{\rm{left}}}^{{{\mathscr{P}}}_{z}}+O{F}_{{\rm{right}}}^{{{\mathscr{P}}}_{z}}}{2}\end{array}$$

where ${{\mathscr{P}}}_{x}$, ${{\mathscr{P}}}_{y}$ and ${{\mathscr{P}}}_{z}$ are the optic flow pooling methods used for lateral control, speed control, and vertical control, $O{F}_{{\rm{left}}}^{{\mathscr{P}}}$ and $O{F}_{{\rm{right}}}^{{\mathscr{P}}}$ are the optic flow pooled using the pooling method ${\mathscr{P}}$ on the left and right side, according to equations (2–5), *OF*_down_ is the ventral optic flow computed according to equation [6], and *OF*_ref_ is a constant reference value. For vertical control–unlike in the uncoupled case (equation [8]) – the ventral flow is compared to a non-constant reference value computed from lateral optic flow.

### Prediction method

Lateral position *y*_pred_, vertical position *z*_pred_, and speed *V*_pred_ are predicted using an iterative gradient descent approach. As a first step, (*y*, *z*, *V*) are given initial values (*y*_0_, *z*_0_, *V*_0_). The initial values are found using a grid search with rough resolution in order to avoid local minima later in the gradient descent algorithm. The initial values are those where the average force applied to an agent while it is flying along the longitudinal axis of the corridor is the lowest.

10 $$(\begin{array}{c}{V}_{0}\\ {y}_{0}\\ {z}_{0}\end{array})={{\rm{argmin}}}_{y,z,V}(\Vert {\int }_{{x}_{{\rm{\min }}}}^{{x}_{{\rm{\max }}}}\overrightarrow{f}(x,y,z,V\mathrm{).}dx\Vert )$$

At the *k*-th iteration, the current lateral position, vertical position and flight speed (*y*_*k*_, *z*_*k*_, *V*_*k*_) define a straight trajectory flown at constant speed along the longitudinal axis *x* of the corridor. In order to know whether this trajectory is an equilibrium point – the predicted trajectory – or whether the agent will be pushed away from this trajectory by the applied forces, the force field is evaluated and summed along the trajectory.

11 $$(\begin{array}{c}d{V}_{k}\\ d{y}_{k}\\ d{z}_{k}\end{array})={\int }_{{x}_{{\rm{\min }}}}^{{x}_{{\rm{\max }}}}\overrightarrow{f}(x,{y}_{k},{z}_{k},{V}_{k}\mathrm{).}dx$$

The result of this operation is an increment in flight speed *dV*, an increment in lateral position *dy*, and an increment in vertical position *dz*, that are used to iteratively update the predicted positions and speed by gradient descent.

12 $$(\begin{array}{c}{V}_{k+1}\\ {y}_{k+1}\\ {z}_{k+1}\end{array})=(\begin{array}{c}{V}_{k}\\ {y}_{k}\\ {z}_{k}\end{array})+(\begin{array}{c}d{V}_{k}\\ d{y}_{k}\\ d{z}_{k}\end{array})$$

The iterative process described in equations [11] and [12] is repeated until a minimum number of iterations are performed and the increments *dV*, *dy* and *dz* are below a small threshold (10^−6^ in our case).

### Prediction error

The predictions are evaluated by comparing them to the recorded bee trajectories to obtain the prediction error *e*

13 $$e=\sqrt{{e}_{x}^{2}+{e}_{y}^{2}+{e}_{z}^{2}}$$

which is computed from the error on flight speed *e*_*x*_, lateral position *e*_*y*_ and vertical position *e*_*z*_

14 $$\begin{array}{rcl}{e}_{x} & = & \sum _{{{\mathscr{C}}}_{\times |\times }}\frac{1}{{N}_{{\mathscr{C}}}}\frac{{V}_{{\rm{pred}}}-{\mu }_{V}}{{\sigma }_{V}}\\ {e}_{y} & = & \sum _{{{\mathscr{C}}}_{\times |\times }}\frac{1}{{N}_{{\mathscr{C}}}}\frac{{y}_{{\rm{pred}}}-{\mu }_{y}}{{\sigma }_{y}}\\ {e}_{z} & = & \sum _{{{\mathscr{C}}}_{\times |\times }}\frac{1}{{N}_{{\mathscr{C}}}}\frac{{z}_{{\rm{pred}}}-{\mu }_{z}}{{\sigma }_{z}}\end{array}$$

where ${{\mathscr{C}}}_{\times |\times }$ indicates each one of the ${N}_{{\mathscr{C}}}$ tested conditions. *V*_pred_, *y*_pred_, and *z*_pred_ are the predicted flight speed, lateral position and vertical position. *μ*_*V*_, *μ*_*y*_ and *μ*_*z*_ are the mean of the measured flight speed, lateral position and vertical position for each condition. *σ*_*V*_, *σ*_*y*_ and *σ*_*z*_ are the standard deviations of the measured flight speed, lateral position and vertical position for each condition.

In equation [14], the error between predicted values and mean of the measured values is divided by the standard deviation of the measured values. This allows us to compare errors on different axes, even though they have different scales and units. In addition, dividing the prediction errors by the standard deviation of the measured data puts more emphasis on predictions in cases where bees behaved in a consistent manner (i.e. low standard deviation) compared to the cases where bees had more variable behaviour (i.e. large standard deviation).

## Supplementary information

Supplementary Material

## Data Availability

The datasets generated during and/or analysed during the current study are available from the corresponding author on request.

## References

[1] Floreano D, Wood RJ (2015). Science, technology and the future of small autonomous drones. Nature.

[2] Egelhaaf M, Kern R (2002). Vision in flying insects. Current Opinion in Neurobiology.

[3] Borst A (2014). Fly visual course control: behaviour, algorithms and circuits. Nature reviews. Neuroscience.

[4] Hassenstein B, Reichardt W (1956). Systemtheoretische analyse der zeit, reihenfolgen, und vorzeichenauswertung bei der bewegungsperzepion des Rüsselkäfers Chlorophanus. Naturforsch.

[5] Franz MO, Krapp HG (2000). Wide-field, motion-sensitive neurons and matched filters for optic flow fields. Biological Cybernetics.

[6] Hyslop A, Krapp HG, Humbert JS (2010). Control theoretic interpretation of directional motion preferences in optic flow processing interneurons. Biological Cybernetics.

[7] Keshavan, J., Gremillion, G., Escobar-Alvarez, H. & Humbert, J. S. A *μ* analysis-based, controller-synthesis framework for robust bioinspired visual navigation in less-structured environments. *Bioinspiration and Biomimetics* **9** (2014). 10.1088/1748-3182/9/2/025011 24852145

[8] Bertrand OJ, Lindemann JP, Egelhaaf M (2015). A Bio-inspired Collision Avoidance Model Based on Spatial Information Derived from Motion Detectors Leads to Common Routes. PLoS Computational Biology.

[9] Srinivasan MV, Lehrer M, Kirchner WH, Zhang SW (1991). Range perception through apparent image speed in freely flying honeybees. Visual Neuroscience.

[10] Gibson JJ (1950). The perception of the visual world. Psychological Bulletin.

[11] Koenderink JJ, van Doorn AJ (1987). Facts on optic flow. Biological Cybernetics.

[12] Portelli G, Ruffier F, Roubieu FL, Franceschini N (2011). Honeybees’ speed depends on dorsal as well as lateral, ventral and frontal optic flows. PLoS One.

[13] Linander N, Baird E, Dacke M (2017). How bumblebees use lateral and ventral optic flow cues for position control in environments of different proximity. Journal of Comparative Physiology A.

[14] Raharijaona, T., Serres, J., Vanhoutte, E. & Ruffier, F. Toward an insect-inspired event-based autopilot combining both visual and control events. *2017 3rd International Conference on Event-Based Control, Communication and Signal Processing, EBCCSP 2017 - Proceedings* (2017).

[15] David CT (1982). Compensation for heigt in the control of groundspeed by Drosophila in a new, ‘Barber Pole’ wimd tunnel. Journal of Comparative Physiology A.

[16] Srinivasan MV, Zhang SW, Lehrer M (1998). Honeybee navigation: Odometry with monocular input. Animal Behaviour.

[17] Baird E (2005). Visual control of flight speed in honeybees. Journal of Experimental Biology.

[18] Baird E, Kornfeldt T, Dacke M (2010). Minimum viewing angle for visually guided ground speed control in bumblebees. Journal of Experimental Biology.

[19] Baird E, Dacke M (2012). Visual flight control in naturalistic and artificial environments. Journal of Comparative Physiology A.

[20] Linander N, Baird E, Dacke M (2016). Bumblebee flight performance in environments of different proximity. Journal of Comparative Physiology A.

[21] Portelli G, Ruffier F, Franceschini N (2010). Honeybees change their height to restore their optic flow. Journal of Comparative Physiology A.

[22] Portelli G, Serres JR, Ruffier F (2017). Altitude control in honeybees: Joint vision-based learning and guidance. Scientific Reports.

[23] Srinivasan MV, Zhang S (2004). Visual Motor Computations in Insects. Annual Review of Neuroscience.

[24] Serres JR, Masson GP, Ruffier F, Franceschini N (2008). A bee in the corridor: Centering and wall-following. Naturwissenschaften.

[25] Srinivasan MV, Zhang SW, Chahl JS, Barth E, Venkatesh S (2000). How honeybees make grazing landings on flat surfaces. Biological cybernetics.

[26] Portelli, G., Serres, J., Ruffier, F. & Franceschini, N. A 3D insect-inspired visual autopilot for corridor-following. In *Proceedings of the 2nd Biennial IEEE/RAS-EMBS International Conference on Biomedical Robotics and Biomechatronics, BioRob 2008*, 19–26 (2008).

[27] Baird E, Boeddeker N, Ibbotson MR, Srinivasan MV (2013). A universal strategy for visually guided landing. Proceedings of the National Academy of Sciences of the United States of America.

[28] Taylor GJ, Luu T, Ball D, Srinivasan MV (2013). Vision and air flow combine to streamline flying honeybees. Scientific reports.

[29] Srinivasan MV, Zhang SW, Chandrashekara K (1993). Evidence for two distinct movement detecting mechanisms in insect vision. Naturwissenschaften.

[30] Rumelhart DE, Zipser D (1985). Feature discovery by competitive learning. Cognitive Science.

[31] Plett J, Bahl A, Buss M, Kühnlenz K, Borst A (2012). Bio-inspired visual ego-rotation sensor for MAVs. Biological Cybernetics.

[32] Linander, N., Dacke, M. & Baird, E. Bumblebees measure optic flow for position and speed control flexibly within the frontal visual field. *Journal of Experimental Biology* 1051–1059 (2015). 10.1242/jeb.107409 25657205

[33] Lecoeur J, Baird E, Floreano D (2018). Spatial Encoding of Translational Optic Flow in Planar Scenes by Elementary Motion Detector Arrays. Scientific Reports.

[34] Zoran, D. & Weiss, Y. Natural Images, Gaussian Mixtures and Dead Leaves. *Advances in Neural Information Processing Systems* 1736–1744 (2012).

[35] Cao, F., Guichard, F. & Hornung, H. Dead leaves model for measuring texture quality on a digital camera. In Imai, F., Sampat, N. & Xiao, F. (eds) *Proc. SPIE 7537, Digital Photography VI, 75370E*, 75370E (2010).

