# Supplementary material for: The role of optic flow pooling in insect flight control in cluttered environments
Source: Sci Rep. 2019 May 22;9:7707. doi: 10.1038/s41598-019-44187-2 (PMC6531491; doi:10.1038/s41598-019-44187-2)
Supplement: Supplementary file 1 — Supplementary Material [file 41598_2019_44187_MOESM1_ESM.pdf]

The role of optic flow pooling in insect flight control in cluttered environments

Julien Lecoeur[1,*], Marie Dacke[2], Dario Floreano[1,+], and Emily Baird[2,3,+]

[1]Laboratory of Intelligent Systems, Institute of Microengineering, École Polytechnique Fédérale de Lausanne, Lausanne, CH-1015, Switzerland
[2]Lund Vision Group, Department of Biology, Lund University, Lund SE-22362, Sweden
[3]Division of Functional Morphology, Department of Zoology, Stockholm University, Stockholm SE-10691, Sweden
[*]To whom correspondence should be addressed. E-mail: julien.lecoeur@epfl.ch
[+]D.F. (Dario Floreano) and E.B. (Emily Baird) share senior authorship.

S1 Detail of prediction errors with uncoupled forward and vertical control

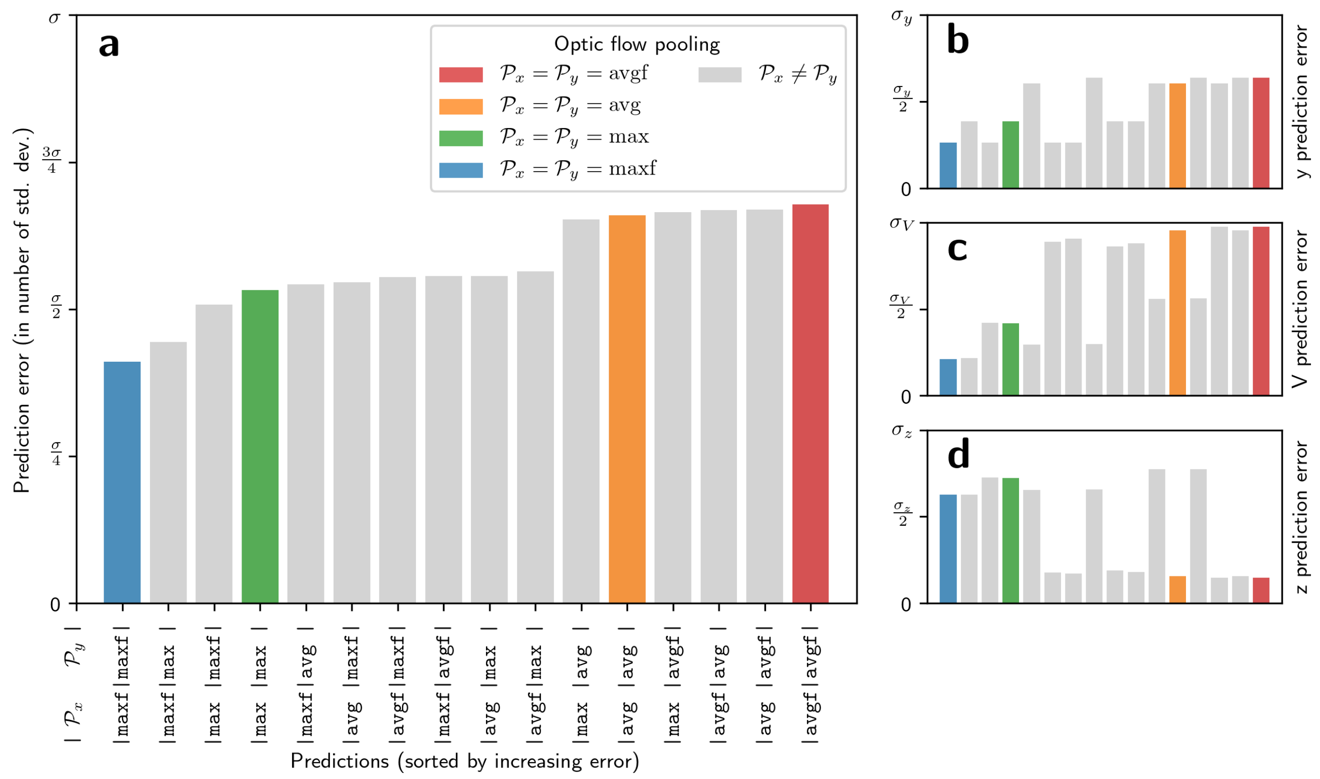

Figure S1. Joint prediction of lateral position, vertical position and speed. Average prediction errors for different optic flow pooling $\mathcal{P}_x$ and $\mathcal{P}_y$. The predictions of flight speed and lateral position are made using different pooling $\mathcal{P}_x$ and $\mathcal{P}_y$ respectively for the control of flight speed and lateral position. The predictions are made according to the control method shown in equation (8). Each prediction is compared with experimental data. The resulting error is scaled according to the standard deviation of the experimental data as shown in equation (14) to allow comparison between values predicted on different axes and units. The scaled errors are then averaged over conditions $\mathcal{C}_{0|0}$, $\mathcal{C}_{0|16}$, $\mathcal{C}_{0|33}$,$\mathcal{C}_{0|100}$, $\mathcal{C}_{16|16}$, and $\mathcal{C}_{33|33}$. computed according to equation (13). (**a**): Average prediction error on all axes. (**b**): Average prediction error for flight speed. (**c**): Average prediction error for lateral position. (**d**): Average prediction error for vertical position.

S2 Detail of prediction errors with coupled forward and vertical control

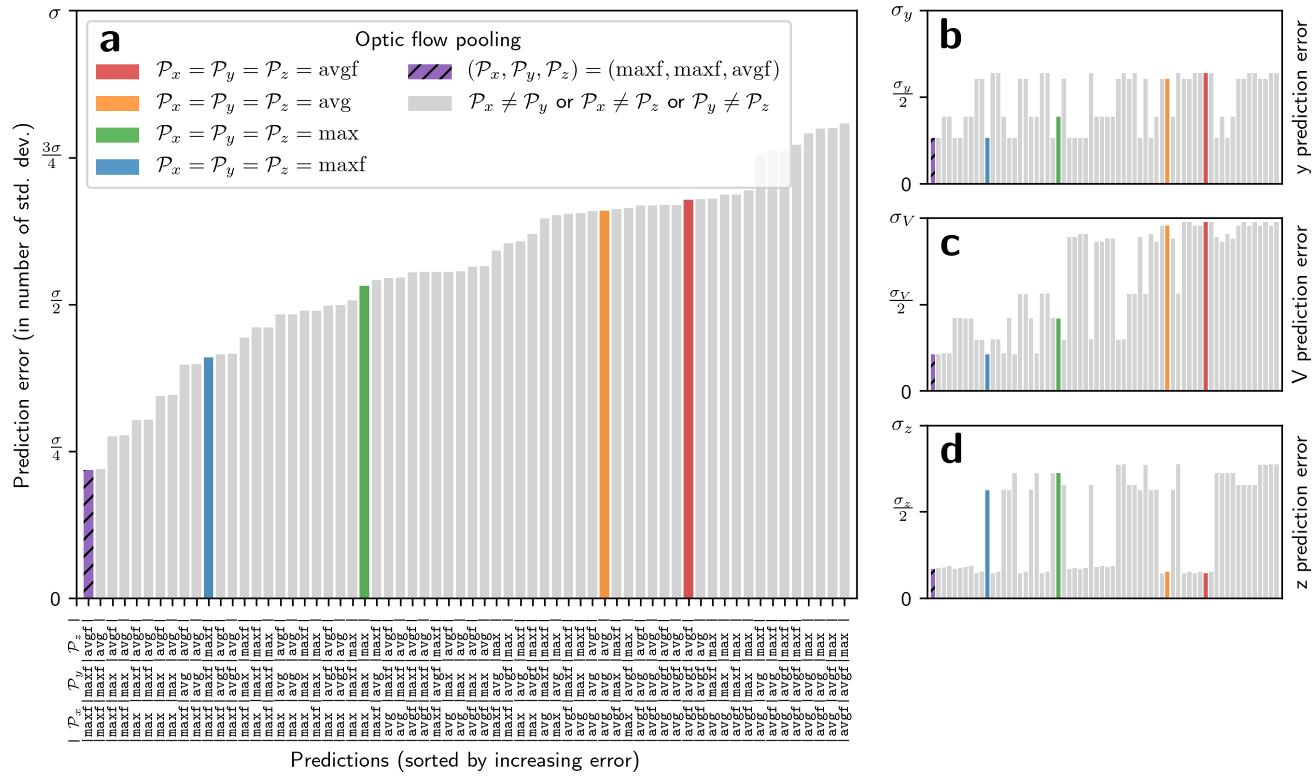

Figure S2. **Joint predictions with coupled vertical and forward control.** Average prediction errors for different optic flow pooling $\mathcal{P}_x$, $\mathcal{P}_y$, and $\mathcal{P}_z$. The predictions of flight speed, lateral position and vertical position are made using different pooling $\mathcal{P}_x$, $\mathcal{P}_y$, $\mathcal{P}_z$ respectively for the control of flight speed, lateral position and vertical position. The predictions are made according to the control method shown in equation (9), where the reference optic flow used for vertical control is not constant, but is instead equal to the optic flow perceived on the left and right sides. Each prediction is compared with experimental data. The resulting error is scaled according to the standard deviation of the experimental data as shown in equation (14), in order allow comparison between values predicted on different axes and units. The scaled errors are then averaged over conditions $\mathcal{C}_{0|0}$, $\mathcal{C}_{0|16}$, $\mathcal{C}_{0|33}$,$\mathcal{C}_{0|100}$, $\mathcal{C}_{16|16}$, and $\mathcal{C}_{33|33}$. computed according to equation (13). (**a**): Average prediction error on all axes.
(**b**): Average prediction error for flight speed. (**c**): Average prediction error for lateral position. (**d**): Average prediction error for vertical position.

S3 Prediction of lateral position alone

Our prediction method can be applied to simpler cases where the agent controls only one of its degrees of freedom, for example its lateral position. The predictions of lateral position only are made using the force field presented in equation (S1).

$$\begin{aligned} f_x^- &= 0 \\ f_y &= OF_{\text{right}}^{\mathcal{P}_y} - OF_{\text{left}}^{\mathcal{P}_y} \\ f_z^- &= 0 \end{aligned} \tag{S1}$$

where $\mathcal{P}_y$ is the optic flow pooling method used for lateral control, $OF_{\text{left}}^{\mathcal{P}}$ and $OF_{\text{right}}^{\mathcal{P}}$ are the optic flow calculated from the pooling method $\mathcal{P}$ on the left and right side. The lateral force f_y has the same formulation as in the cases where three degrees of freedom of the agent are controlled (equation [8, 9]), but the forward force f_x^- and the vertical force f_z^- are null.

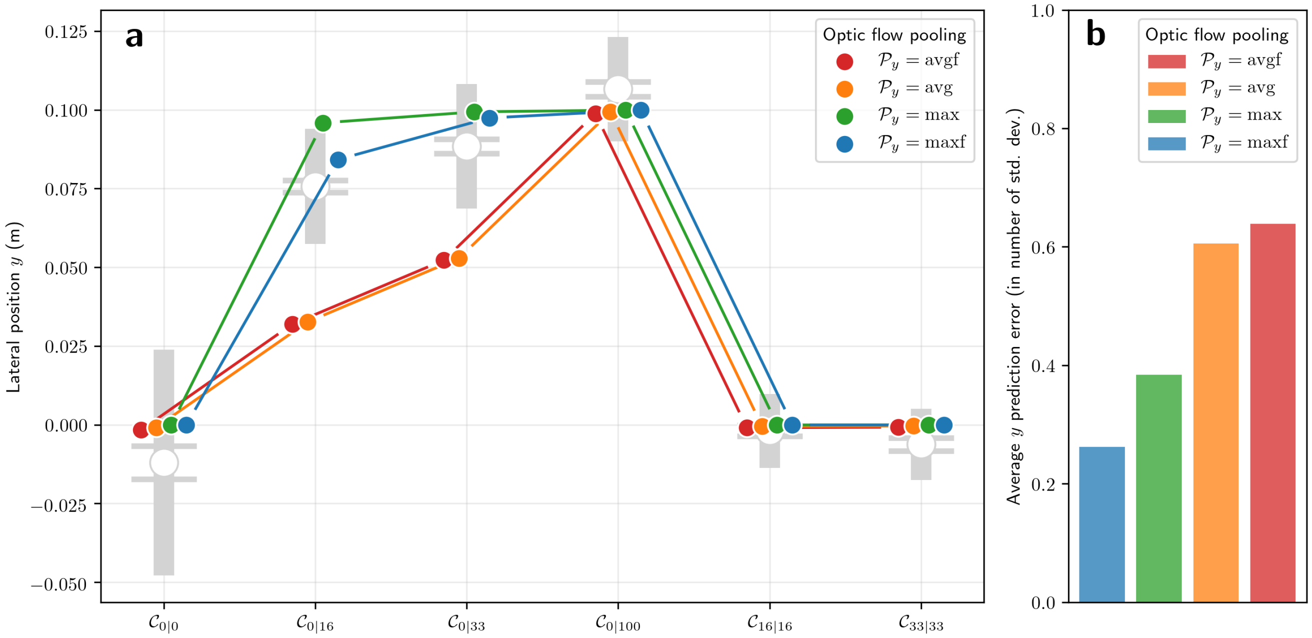

Figure S3. Predicted lateral position. The experimental measurements of bumblebee lateral position (y) are compared to predicted values. Predictions are made according to the control method described in equation (S1), with a single optic flow pooling $\mathcal{P}_y$. Predictions are made for four optic flow pooling methods `avg`, `avgf`, `max`, and `maxf` described in equations (2, 3, 4, 5). The best prediction for lateral position is made with `maxf` pooling. The predictions are the same as when flight speed and vertical position are also predicted (Fig. 3 and Fig. 5). (**a**): Measured and predicted lateral position for each test condition. Measurements are displayed as grey open circles. Similarly to Fig. 2, the horizontal lines on the error bars denote standard error of the mean. The uncapped bars denote the standard deviation. Predictions are displayed as solid coloured circles. Note that predictions are shifted horizontally for better presentation, but they are not shifted vertically. (**b**): Average prediction error for lateral position. Each prediction is compared with experimental data. The resulting error is scaled according to the standard deviation of the experimental data as shown in equation (14). This scaling allows us to compare prediction errors between conditions where bees controlled their flight with variable precision. The scaled errors are then averaged over conditions $\mathcal{C}_{0|0}$, $\mathcal{C}_{0|16}$, $\mathcal{C}_{0|33}$,$\mathcal{C}_{0|100}$, $\mathcal{C}_{16|16}$, and $\mathcal{C}_{33|33}$.

S4 Illustration of the prediction method for `maxf` optic flow pooling

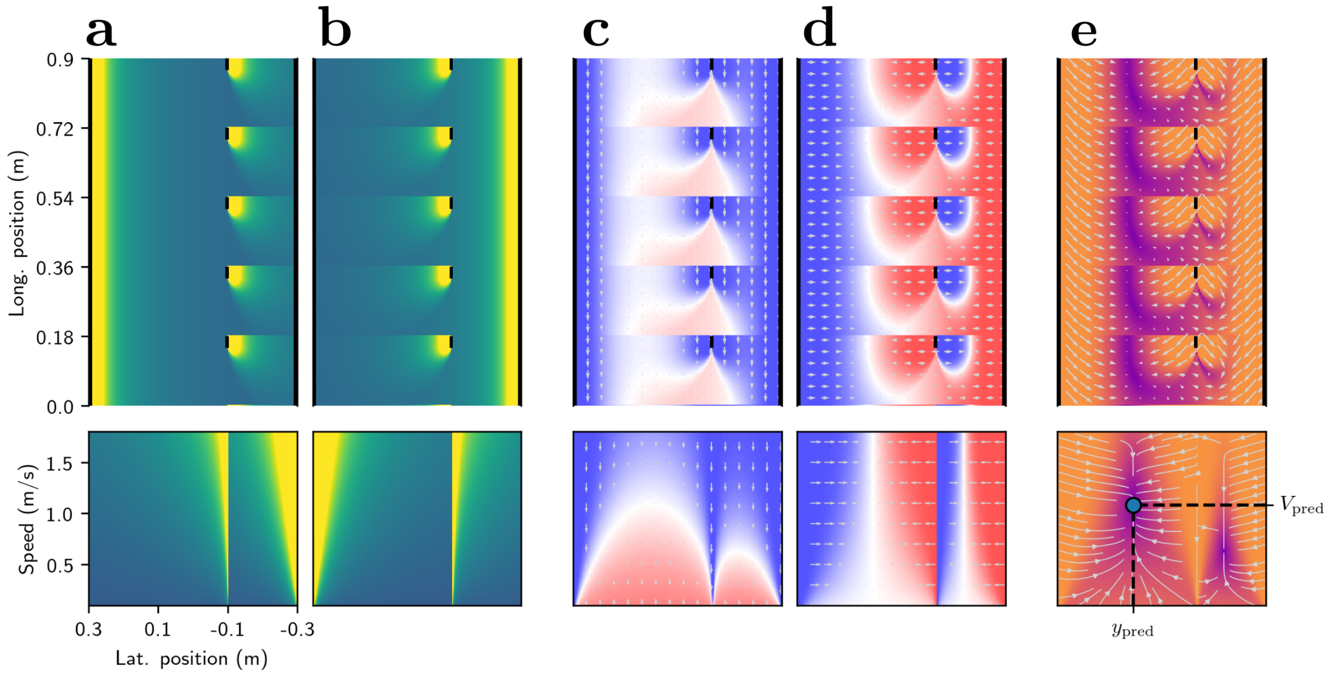

Figure S4. Illustration of the prediction method for `maxf` optic flow pooling. (**a** and **b**): Optic flow amplitude on the left side $OF_{\text{left}}^{\mathcal{P}_{\texttt{maxf}}}$ and on the right side $OF_{\text{right}}^{\mathcal{P}_{\texttt{maxf}}}$, computed according to equation [5]. Blue represents low optic flow amplitude, yellow represents large optic flow amplitude. (**c**): Forward force f_x used to control flight speed and computed according to equation [8]. Blue represents negative f_x values (deceleration) and red represents positive f_x values (acceleration). (**d**): Lateral force f_y used to control lateral position and computed according to equation [8]. Blue represents negative f_y values (rightward force) and red represent positive f_y values (leftward force). (**e**): Combined force field $\vec{f}$. The white arrows represent the trajectories of an agent driven by the force field. The prediction is found by following the white arrows by gradient descent (see equation (12)).
